# Clinical evaluation of silicone gel in the treatment of cleft lip scars

**DOI:** 10.1038/s41598-018-25697-x

**Published:** 2018-05-09

**Authors:** Chun-Shin Chang, Christopher Glenn Wallace, Yen-Chang Hsiao, Jung-Ju Huang, Zung-Chung Chen, Chee-Jen Chang, Lun-Jou Lo, Philip Kuo-Ting Chen, Jyh-Ping Chen, Yu-Ray Chen

**Affiliations:** 1 Department of Chemical and Materials Engineering, College of Engineering, Chang Gung University, Taoyuan, Taiwan; 2 Craniofacial Research Center, Department of Medical Research, Department of Plastic & Reconstructive Surgery and Department of Craniofacial Orthodontics, Chang Gung Memorial Hospital, Taoyuan, Taiwan; 3 Department of Plastic Surgery, Royal Devon & Exeter Hospital, Exeter, EX1 1AP United Kingdom; 4 Graduate Institute of Clinical Medical Sciences, School of Medicine, Chang Gung University, Taoyuan, Taiwan; 5 Department of Plastic Surgery, Xiamen Chang Gung Hospital, Xiamen, China

## Abstract

Upper lip scars are at risk of hypertrophy. Our center therefore uses microporous tape and silicone sheeting for postoperative scar care following cleft lip repair. However, some babies have previously ingested their silicone sheeting, which has the potential for respiratory compromise or gastrointestinal obstruction. Self-dry silicone gel is reportedly also effective for preventing hypertrophic scars. Hence, we sought to test whether silicone gel, which cannot be ingested whole, might be non-inferior to silicone sheeting for controlling against upper lip scar hypertrophy. This was a mixed prospective and retrospective case-controlled clinical trial involving patients undergoing unilateral cleft lip repair, 29 of whom received standard postoperative silicone sheeting (control group) and another 33 age-matched consecutive patients who received self-dry silicone instead. The Vancouver scar scale, visual analogue scale and photographically assessed scar width assessments were the same in both groups at six months after surgery. In conclusion, silicone gel appears to be non-inferior to silicone sheeting for postoperative care of upper lip scars as judged by scar quality at six months, but silicone sheeting has the safety disadvantage that it can be swallowed whole by babies. It is thus recommended that silicone gel be used for upper lip scar management in babies.

## Introduction

Cleft lip/palate is the most common craniofacial anomaly in humans. Lip repair is one of the most important reconstructions for these patients, and is performed at around 3 months of age at our institution. Although the cheiloplasty scar is unavoidable and permanent, every possible measure should be considered to optimize its functional and aesthetic outcome, since the scar can be a lifelong social stigma of a cleft lip operation. Hypertrophic scarring can highlight the scar even further, and is a recognized negative outcome for cheiloplasty. Moreover, with an incidence as high as 36.3%^1^, hypertrophic scars are more common in Asian-Orientals compared to Caucasians.

The population treated at our institution is almost entirely Oriental (Taiwanese). Our patients’ intrinsic higher risk of hypertrophic scarring has led us continuously to try to improve scar quality for them. In 2011, we started a double-blinded, randomized, vehicle-controlled, prospective clinical trial to evaluate whether the injection of botulinum toxin A into the orbicularis oris muscle could improve the quality of the cleft lip scar^2^. Our results revealed that botulinum toxin injections into the subjacent orbicularis oris muscle produced narrower cheiloplasty scars, but provided no additional benefits in terms of scar pigmentation, vascularity, pliability or height. During that study, the parents of 14% (4/29) of the babies within the control group reported that their baby had tried, albeit unsuccessfully, to ingest the silicone sheet at night. This caused us to question the safety of silicone sheeting on the upper lip in babies since attempted ingestion can also lead to aspiration.

Silicone is known to be effective for treating and/or preventing hypertrophic scarring^3–7^. Silicone gel has been shown to prevent hypertrophic scars in median sternotomy wounds^8^. We therefore conducted this clinical trial to evaluate whether post-operative use of silicone gel was non-inferior to silicone sheet for preventing hypertrophy of unilateral cleft lip repair scars.

## Results

Twenty-eight patients in the experimental group completed the study, three patients having been excluded because they did not return to the six month follow up clinic and two others because they did not comply with the postoperative scar care regimen. Patients’ demographics and cleft gap before surgery are listed in Table 1. No post-operative complications occurred (infection, bleeding, wound dehiscence).

**Table 1:** The patients’ demographics and the cleft gap width before surgery.

	Study group	Placebo group	
Patient number	28	29	
Age (months)	3.36 ± 0.61	3.17 ± 0.25	T test P = 0.152
Sex (Male:Female)	(17:11)	(19:10)	Chi Square test P = 0.707
Cleft side (Left:Right)	(14:14)	(24:5)	Chi Square P = 0.009
Cleft gap (mm)	0.67 ± 0.34	0.61 ± 0.25	T test P = 0.429

VSS scores in the experimental and control groups were similar (2.2 ± 1.74 versus 2.76 ± 1.44 for experimental and control groups, respectively; p = 0.189). VSS interobserver consistency was high (cronbach α = 0.899). VAS scores in the experimental and control groups were similar (7.41 ± 0.90 versus 7.19 ± 0.95; p = 0.374) and VAS interobserver consistency was high (cronbach α = 0.855). Photographically-assessed scar widths in both groups were similar at both the first (0.4 ± 0.15 mm versus 0.45 ± 0.11 mm for experimental and control groups, respectively; p = 0.205) and second points (0.49 ± 0.37 mm versus 0.47 ± 0.13 mm; p = 0.736). Interobserver consistency was high for photographic measurements (r = 0.965 and p < 0.001 for first points and r = 0.993 and p < 0.001 for second points).

## Discussion

A barely noticeably scar is desired by almost all patients who have undergone cleft lip repair. The goal of primary cleft lip repair is therefore to achieve a normal looking and functioning lip and nose, leaving minimal visible stigmata^9,10^. However, this scar is at increased risk of scar hypertrophy due to the repetitive movements of the upper lip that have a tendency to distract the wound edges whilst it is healing^1^.

Silicone sheeting is an adhesive, soft and semi-occlusive dressing that has been a popular topical treatment for hypertrophic and keloid scars for decades^11^, supported by systematic review of the evidence for its effectiveness^12^. Side effects include rashes and skin breakdown, and usability problems include loss of adhesiveness^13^. It is recommended as a first line preventative, or treatment, for unfavorable scarring in Asian patients, and we prescribe it for our lip repair patients as part of their postoperative scar care protocol that also includes six months of microporous tape traversing the upper lip from cheek-to-cheek during the day and silicone sheeting at night supported by a shorter tape^8,14,15^.

However, over the years, some babies have managed successfully to ingest their silicone sheet at night, which has caused us to question the use of this silicone preparation in this setting. Approximately 40% of foreign body ingestions by children go unwitnessed, and commonly these involve small toys^16^. Fortunately, most swallowed objects pass through the entire digestive tract uneventfully^17^. The silicone sheets used for the babies measured approximately 1 × 1 cm, which is not dissimilar to dimes, or other coins, that have been reported as symptomatic esophageal foreign bodies in children^18^. Reported hazards of retained coins range from esophageal perforation, esophageal stricture, and respiratory distress^19^. Our patients use the silicone sheet during an age range of 3 to 12 months of age, when the caliber of the esophagus is smaller and the risk for respiratory aspiration exists. Babies who have swallowed their silicone sheets overnight following cleft lip repair at our institution have caused considerable worry for their parents, who have brought their babies back to hospital for emergency room assessments. Identifying whether or not the silicone sheet has been swallowed or aspirated has also proven difficult, since the sheeting is radiolucent. Although rare, ingestion or aspiration of the silicone sheet can cause intestinal or respiratory compromise, just like other ingested foreign bodies, and can lead to a pediatric emergency department presentation^20^.

**Figure 1:**
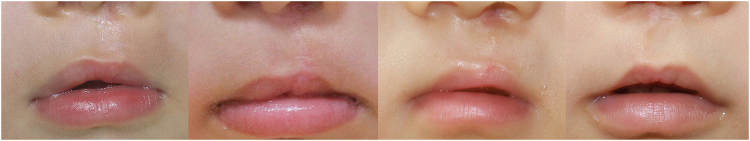
Babies from the study group (silicone gel group) showing similar quality scars to the control group.

**Figure 2:**
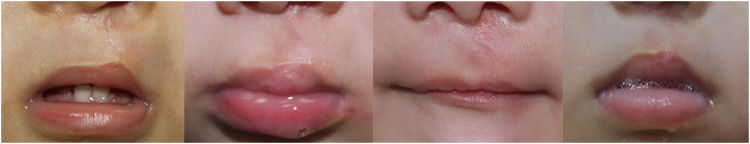
Babies from the control group (silicone sheet group).

The present study introduced commercially available self-dry silicone gel containing vitamin C to our center^21^. Silicone gel reportedly has similar efficacy to silicone sheets^22^, but the critical advantage of gel for babies is that, unlike sheeting, gel is not a solid foreign body that can be swallowed whole. The gel dries within 3 minutes and parents reported no difficulties during application. According to this present study, neither form of silicone offered a scar quality advantage over the other, however the silicone sheet has the safety disadvantage (Figs 1 and 2).

In this study, scar width measurements were performed photographically. We previously used ultrasound to measure scar widths in adults^23^; however, we found that this was not possible in babies as the investigation caused them to cry and therefore move, which caused difficulties with taking accurate measurements.

The limitations of the present study merit emphasis. First, this study is inferior in methodology to a randomized controlled study with blinding, which is warranted in the future and from which firmer conclusions about non-inferiority could be more confidently drawn. Second, the control group in the present study was obtained from a previous randomized controlled study^2^, as was approved by the Institutional Review Board; firmer conclusions about non-inferiority could be drawn, however, if the control group instead underwent recruitment specifically for the present study. Third, it was surprising to find that, compared to the control group, there were more right sided cleft lip repairs in the study group (Table 1), however, all other factors were similar between the groups: operative age, sex, cleft gap and surgical techniques.

In conclusion, silicone gel is recommended over silicone sheeting for postoperative care of upper lip scars in babies, as it appears to be non-inferior to silicone sheet in terms of scar outcome but has the advantage that it cannot be ingested whole as a foreign body.

## Methods

This is a mixed prospective and retrospective case-controlled clinical trial primarily designed to compare scar quality after primary cleft lip repair with post-operative use of self-drying silicone gel versus with post-operative use of silicone sheeting. There was no blinding and no randomization.

### Ethics and Groups

The study was ethically approved by the institutional review board of Chang Gung Memorial Hospital (IRB No 102-4719b) on the 28^th^ January 2014 and was registered at ClinicalTrial.gov (NCT03314090) on the 16^th^ October 2017. All methods were performed in accordance with the relevant guidelines and regulations. Recruitment for the study group commenced on the 12^st^ August 2014 and the final patient was recruited on the 7^th^ June 2016. Follow-up for the final patient completed on the 29^th^ November 2016. All patients in the study group provided IRB-approved fully informed written consent for inclusion into this study.

The control group provided informed consent for our previous study (Botulinum Toxin to Improve Results in Cleft Lip Repair; IRB No 101-3009 C)^2^; their inclusion as controls for this study with new consents was approved by the Institutional Review Board (IRB No 102-4719b). The control group consisted of 29 patients who were treated using the current established protocol for scar care following cheiloplasty^2^. This involved microporous tape placed across both cheeks and spanning the upper lip during daytime, and silicone sheets fixed with a shorter length of microporous tape (that did not span the cheeks) at night. This continued strictly for 6 months.

The Study group consisted of another 33 consecutive age-matched patients with unilateral cleft lip, whose postoperative scar care was exactly the same except the silicone sheet was replaced with silicone gel (Dermatix Ultra, Menarini, Singapore), which was applied twice per day. The parents (or caregivers) were instructed to apply silicone gel (the amount being similar in size to a grain of rice) along the upper lip scar from the nostril base to the vermillion, avoiding the wet mucosa.

Inclusion criteria were: 1. Baby born with cleft lip planned for primary lip repair around 3 months of age, 2. Written informed consent for the study provided by the parent/guardian. Exclusion criteria were: 1. presence of other craniofacial anomalies; 2. lack of signed informed consent from the parent/guardian.

### Patient sample size

The sample size was calculated based on the results of previous study. The Vancouver Scar Scale (VSS) presented a mean score of 2.76 ± 1.44. If treatment could improve VSS score by 1, which was considered clinically significant, approximately 33 patients for the study group would have been necessary to provide a result with a real significance (using the same standard deviation, considering the standard type 1 α error of 0.05 and a power of 0.8). For scar width, the first and second points presented a mean score of 0.45 ± 0.11 and 0.47 ± 0.13. If a scar width difference of 0.1 mm was considered clinically significant, approximately 19 and 27 patients for the study group would be necessary to provide a result with a real significance (using the same standard deviation, considering the standard type 1 α error of 0.05 and a power of 0.8). The sample size was calculated as 33 patients for the control group.

### Data sharing statement

The data can be accessed: https://www1.cgmh.org.tw/intr/intr2/c32540/en/clinical_trials_studies.html.

Extra data is available by emailing: plastreconst@gmail.com.

### Primary Cheiloplasty

After pre-surgical nasal-alveolar molding, cheiloplasty was performed around 3 months of age using a Mohler rotation advancement method as previously described. The nasal floor was closed with a CM-flap, L-flap and inferior turbinate flap. The columella base and the caudal philtrum were reconstructed with a C-flap. The lateral orbicularis oris muscle was released and sutured to overlap the medial orbicularis oris muscle such that the main tension of closure was sustained by the muscle repair, leaving mild skin laxity. The skin was closed in two layers: subcutaneous closure with 5-0 Polydioxanone (PDS II; Ethicon/Johnson-Johnson, New Brunswick, New Jersey) and skin closure with 7-0 polyglactin (Vicryl; Ethicon/Johnson-Johnson, New Brunswick, New Jersey). Skin sutures were removed on the sixth postoperative day and the patients were followed up every 1–2 months. During each follow up, parents were asked by open questions how they had cared for the baby’s scar to assess compliance.

### Measurement

Vancouver Scar Scale (VSS): At six months, two plastic surgeons assessed the scars using the VSS and the mean score calculated.

Visual Analogue Scale (VAS): Scar quality was assessed by five independent observers (two plastic surgeons and three laypersons) using a VAS with 10 grades: 0 represented the worst possible scar outcome and 10 the best possible scar outcome.

Scar width measurement: A standard frontally oriented photograph was taken with a surgical ruler placed on the lower lip at the six-month follow up clinic. The scar width measurements were obtained from the photographs (using the surgical ruler as the reference) by two independent raters and means calculated. A commercial photograph program for scar width measurement was utilized (Photoshop CS5 extended version 12.0; Adobe Systems Inc, San Jose, California). Scars were measured at two points: the First Point was 1 mm above the white roll; the Second Point was 1 mm below the C-flap suture line.

### Statistical analyses

Statistical analyses were conducted using SPSS software (version 17.0; IBM Corporation, NY, USA). Cronbach α was used for inter-observer reliability of the VSS and VAS. Pearson Correlation test was used for inter-observer reliability of photographic measurements. Differences between VSS scores, VAS scores and scar widths were compared and the independent t-test was used to compare both groups. The Chi square test was used to compare demographic data. Statistical significance was defined if p was less than 0.05.
